# An Affordable Method for Evaluation of Ataxic Disorders Based on Electrooculography

**DOI:** 10.3390/s19173756

**Published:** 2019-08-30

**Authors:** Alberto López, Francisco Ferrero, Octavian Postolache

**Affiliations:** 1 Departamento de Ingeniería Eléctrica, Electrónica, Computadores y Sistemas, Universidad de Oviedo, Campus de Gijón, 33204 Gijón, Spain; 2 Instituto de Telecomunicações, Av. Rovisco Pais, 1, 1049-001 Lisboa, Portugal; 3 ISCTE-Instituto Universitario de Lisboa, Av. das Forças Armadas, 1649-026 Lisboa, Portugal

**Keywords:** ataxic disorders, electrooculogram (EOG), electrooculography, eye movement, signal processing

## Abstract

Ataxias are a group of neurodegenerative disorders characterized by cerebellar dysfunction that cause irregularities in the rate, rhythm, amplitude, and force of voluntary movements. The electrooculogram (EOG) may provide clues about ataxic disorders because most of these patients have difficulty with visual tracking and fixing their gaze. Using electrodes, EOG records the biopotentials generated by eye movements. In this paper, three surface electrodes are placed around the eye socket, and the biopotentials generated by eye movements are acquired using a commercial bioamplifier device. Next, the signals are sent to the computer to be digitally processed to extract the rate of saccades as well as the delay and deviation of the gaze in response to a stimulus. These features are analysed in a novel software application designed to help physicians in evaluating ataxia. After applying several tests to both healthy and ataxia-affected patients, differences in EOG results were found. The evaluation of the reliability of the designed software application is made according to three metrics: sensitivity, specificity, and accuracy. The results indicate the proposed system’s viability as an affordable method for evaluation of ataxic disorders.

## 1. Introduction

Ataxias are a group of neurodegenerative disorders characterized by cerebellar dysfunction that cause irregularities in the rate, rhythm, amplitude, and force of voluntary movements, especially at initiation and termination of motion, and result in irregular trajectories in limbs. Ataxia disorders are divided into two subgroups: hereditary and nonhereditary. Nonhereditary ataxia develops like Parkinson’s disease, but it could be due to an accident that has injured the nervous system. The cerebellum and brain stem are progressively degenerate, but the progression rate varies considerably among different patients. On the other hand, hereditary ataxias can present autosomal-dominant, autosomal-recessive, recessive X-linked, and mitochondrial inheritance patterns. Each of these inheritance patterns shows an enormous genotypic and phenotypic heterogeneity, which makes it difficult to diagnose based on the clinical observation [1]. In both ataxia subgroups, symptoms are characterized by an incoordination in limb movement and a difficulty to speak, swallow, and maintain balance. The diagnosis is made after ruling out other causes that present similar symptoms.

More than 100 different hereditary ataxia disorders have been identified in the literature and are classified into three subgroups: autosomal dominant cerebellar ataxia, recessively inherited ataxias and maternal inherited ataxias (X-linked and mitochondrial). Autosomal-dominant cerebellar ataxia has a prevalence of 1–5 in 100,000 and includes 40 spinocerebellar ataxias (SCAs). SCA-2 represents 13% to 18% of dominant ataxias worldwide [2,3]. The most common of the recessive ataxias is Friedreich’s ataxia with a prevalence of 1 in 30,000–50,000. It often starts before the age of 25 [4]. Recessive X-linked ataxias are a group of disorders in which the hallmark is a cerebellar defect (hypoplasia, atrophy, or dysplasia) visible on brain imaging, which is caused by gene mutations or genomic imbalances on the X chromosome. It has a prevalence of 1 in 4000–5000 live births [5]. The last subgroup of hereditary ataxias, mitochondrial, is a chronic illness that can be present at birth or develop later in life. More than 20 subtypes of this disease can be distinguished. It is estimated to have a prevalence of ~12.5 per 100,000 in adults and ~4.7 per 100,000 in children [6].

Ataxia is a chronic disorder that currently has no cure, but early diagnosis is important for maintaining a good quality of life. The ataxic disorders can be evaluated knowing the neurological history together with neural imaging (magnetic resonance imaging [MRI] or computed tomography [CT] of the brain or spine) and electrophysiologic studies (electromyogram [EMG], visually evoked response [VER] test, brainstem auditory evoked response [BAER] test, and electroencephalogram [EEG]). These techniques can detect the localization of the process and often its etiology [1]. However, evaluation devices, based on the premise of most patients affected by ataxia disorders have difficulty with visual tracking and in fixing gaze on a stimulus, has not yet been developed. Ataxia patients’ visual function may be intact, but their oculomotor function is dysfunctional due to the inability of the cerebellum to coordinate movements [7,8]. Therefore, the EOG could provide clues about ataxic disorders by recording the biopotentials generated by the eye movements. Nowadays, the EOG is widely used in the clinical environment as support for diagnostic processes or for the design of healthcare systems [9,10,11,12,13,14,15,16].

**Figure 1:**
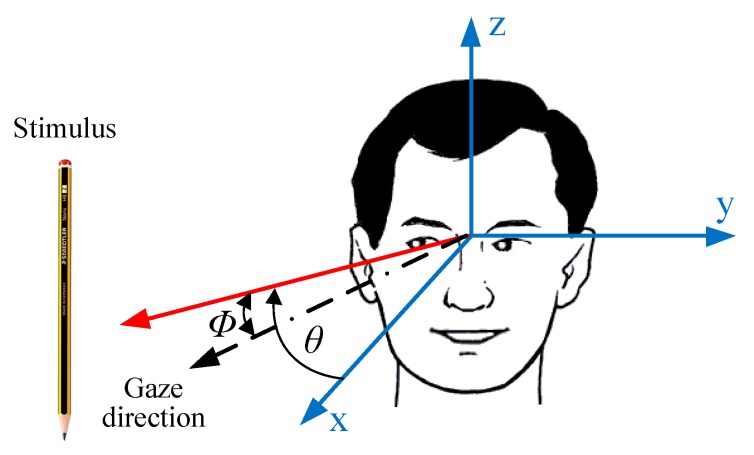
Spatial representation of eye movements to a stimulus.

Figure 1 shows a typical clinical test in which the physician moves a stimulus from left to right and the patient must follow it with his or her eyes. The angle ϕ represents the degree of advancement or delay that occurs when the stimulus moves an angle θ with respect to the coordinate axis. Based on this simple idea, the aim of this study is to develop an EOG-based system to identify abnormalities in the oculomotor system of patients affected by ataxia. To do that, three surface electrodes are placed around the eye socket, and the biopotentials generated by the eye movements are acquired using a commercial bioamplifier device. The signals are then digitally processed to extract eye movement features: rate of saccades and delay and deviation in response to a stimulus. These features are analysed in a computer application designed to help physicians identify an early ataxic disorder.

The remainder of the article is structured as follows: Section 2 covers related work. Section 3 exposes the fundamentals of electrooculography. Section 4 describes the signal acquisition and processing features. The tests conducted are described in Section 5. Finally, Section 6 draws the conclusion.

## 2. Related Work

In recent years, many studies have been published about the evaluation of ataxic disorders. In this section, some of the most relevant ones are briefly discussed. Reference [17] compares two machine learning methods (multilayer perceptron and random forest) to solve the saccade classification problem of EOG signals measured in patients with SCA-2. However, the small number of samples, from six patients, to feed the algorithms without including healthy people to serve as controls calls into question the validity of the results obtained using this diagnostic technique.

The aim of the work presented in [18] is to identify diagnostic features of SCA-6 patients by studying the horizontal vestibulo-ocular reflex (VOR). Even though this technique allows researchers to understand the pathophysiology of the VOR due to cerebellar Purkinje cell degeneration, the results are inconclusive and difficult to extrapolate to other types of ataxia.

Quantitative evaluations of ataxia for arm movement are reported in [19,20,21,22]. These evaluations are limited to movement kinematics, which cannot specify causal muscle activities due to the redundancy of the musculoskeletal system. A method for quantitatively evaluating of ataxic disorders, based on an estimation of the wrist joint torque from EMG signals, is expounded in [23,24,25]. Following the same idea, an automated system to replace the conventional finger chase test, which is performed manually as part of the neurological examination, is presented in [26]. This approach can capture the disability and provide an objective measurement of cerebral ataxia that affects upper limb function. However, it could be a better choice to directly analyse the ocular movement performed to carry out these follow-up activities.

In Refs. [27,28], a triaxial accelerometer is used for the clinical assessment of ataxic patients. A miniature accelerometer was attached to a specified anatomical site, such as the wrist, hand, ankle, or foot. Various parameters obtained by the accelerometer can be sensitive and used as objective markers for the assessment and follow-up of standing and gait impairment in ataxic patients. Triaxial accelerometers obtain more parameters regarding motor function than do force plates or a stabilometer and are also used for ataxia evaluation [29,30]. However, their field of application is too broad for all types of SCAs as well as any multiple system atrophy. It is not only focused on patients with ataxia disorders.

The integration of wearable and wireless inertial body sensors with machine learning has also been investigated to offer the capacity to diagnose neurological disorders involving the gait of people with Friedreich’s ataxia [31]. However, the accuracy obtained in this study (around 70%) invites researchers to examine simpler and more reliable techniques.

## 3. Electrooculography

**Figure 2:**
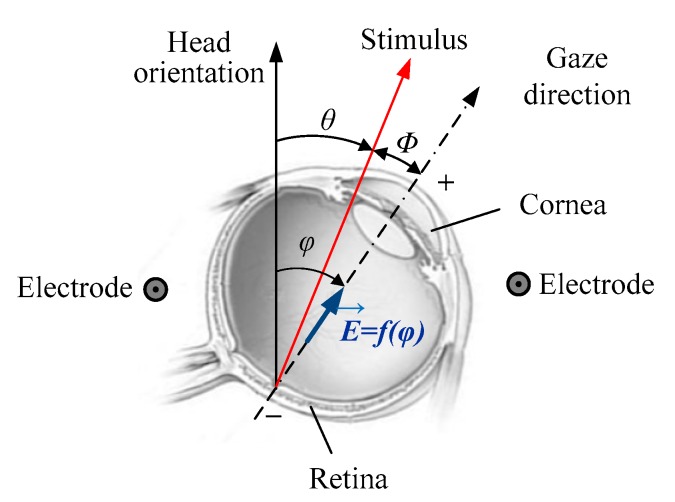
Eyeball modelled as a dipole and angular deviation of the eye movements in response to a stimulus. The vector $\vec{E}=f\left(\varphi \right)$ represents the electric field dependent on the eye rotation angle.

Because of its affordability and accuracy, electrooculography is a well-known technique for measuring and tracking eye movements. It is based on the foundation that the eye can be modeled as an electric dipole, being the cornea electrically positive in reference to the electrically negative retina, so when an eye movement occurs, a differential potential results that relates to the magnitude of rotation. The amplitude obtained depends on the angle through which the eyeball is moved. The recording of this potential is made through surface electrodes and is called EOG. When the eyes move to either side, the voltage goes positive or negative (depending on electrode placement) and remains zero when the eyes look straight. Figure 2 shows a simple representation of this feature.

The voltage amplitude for the horizontal and vertical eye movement is up to 16 µV and 14 µV, respectively, for each degree of eye movement. This behavior is considered nearly linear in the range ±50° for horizontal and ±30° for vertical eye movements [32,33,34]. The recording of the dipole changes requires placing small surface electrodes on the skin around the eyes. There are different possible electrode configurations for different purposes [35]. The recorded signals depend on the electrode setup chosen, because the signal amplitude is positive when the eyes are moving toward the positive electrode and negative when moving toward the negative electrode. The typical EOG amplitude ranges from 0.05–3.5 mV in humans, but this varies with the environmental conditions and in cases of any eye disease [36]. The bandwidth is between dc and 50 Hz, but the frequencies of interest are in the range of 0.1–40 Hz [37].

**Figure 3:**
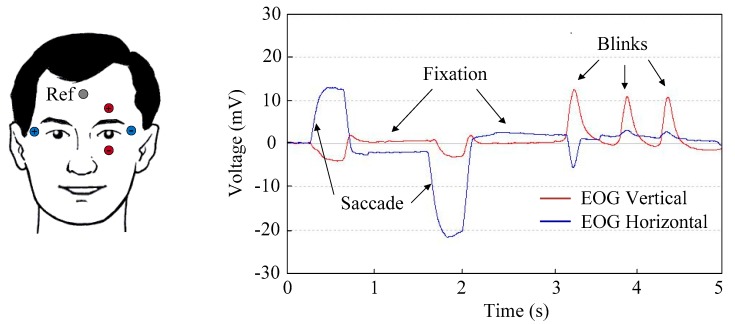
EOG showing saccadic and fixation eye movements as well as blinks. Red and blue waveforms represent the vertical and horizontal potentials, respectively. They are acquired by the electrodes of the same color. The gray electrode is the reference electrode.

The most important patterns that can be identified in the EOG are saccades, fixations, and blinks. Saccadic movements are the most important for this work because they represent the voluntary and simultaneous movement of both eyes. Patients affected by ataxia cannot stare at an object; as a result, involuntary saccades will appear. The peak amplitude depends on the angular stroke of the saccade. Fixations are the state during which the gaze is focused on a specific location [32,33,34,37]. During fixations on an object in the environment, it is possible to discover micromovements of an involuntary nature (drifts and flicks) that have amplitude around 1°. It is also possible to discover tiny movements of tremor, or vibration of the eye, with frequencies between 30 and 150 Hz. Blinks can be involuntary and voluntary. The latter is related to monocular blinks and has 10 to 20 times greater amplitude than involuntary blinks, which is related to ordinary mutual blinking [38,39,40,41]. Figure 3 shows the typical patterns that can be identified in an EOG obtained by recording vertical and horizontal eye potentials using a commercial bioamplifier.

To evaluate possible incoordination in the eye movement that would indicate an ataxic disorder, two characteristics should be observed in patients [1,7,8]: (1) saccadic eye movement tracking moving objects; and (2) capacity to fixate on an object at rest. To evaluate these two characteristics, it is necessary to know that there are two types of eye movement that coordinate vision to track objects: rapid eye movement (REM) and slow eye movement (SEM). REM governs eye movements made to find new objects in the eye’s visual field. It is a fast angular displacement in which the eyes move in a series of small, jerky movements. These movements are so fast that the eye can scroll 10° in just 45 ms. They can be voluntary, such as when reading, or involuntary, such as when an object suddenly enters a person’s visual field. SEM forms a uniform system of chase to adjust the position and speed of the eye with the path and speed of the stimulus. SEM quickly compensates for possible errors of focus in fovea. The speed of such movement is not voluntary and depends on the stimulus [42].

## 4. Materials and Methods

**Figure 4:**
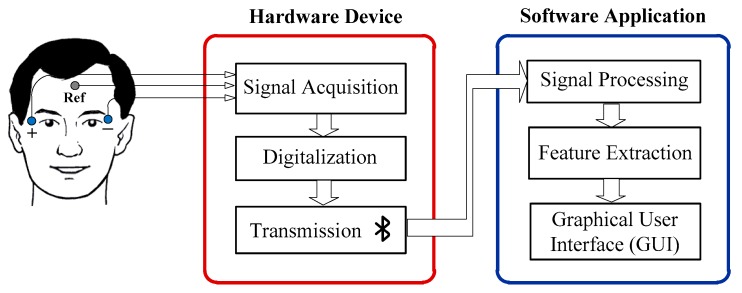
Flow diagram of the main tasks implemented for ataxia evaluation.

The proposed EOG-based system for ataxia evaluation consists of a hardware device (bioamplifier) for recording the EOG signals and a software application to process those signals. The patient’s eyes must follow an image that appears on the computer’s screen as a stimulus. Figure 4 shows a flow diagram of the main tasks to be made for evaluation. The following subsections are devoted to explaining this flow diagram, starting with the electrode placement.

### 4.1. Hardware Device

Three electrodes are placed on either side of the eyes to pick up the biopotentials generated by the motion of the eyeball. Two of them (+, −) are used to achieve the derivation of the EOG signal of the horizontal eye movement, indicated by the y-axis in Figure 1. The third electrode (Ref), which is placed on the forehead, senses the common mode voltage on the body to separate it from the acquired EOG signal. Electrodes should be securely attached to skin to avoid artefacts of movements. Placing them above bony structures where there is less muscle mass can reduce unwanted motion artefacts and EMG interference [35]. By recording only the horizontal eye movement, artefacts due to blinking are substantially reduced, as shown in Figure 3. Ag/AgCl electrode gel is also of considerable importance in maintaining a high-quality interface between the electrodes’ metal and the skin.

To record and send the EOG signal via Bluetooth to the host computer, a commercial bioamplifier from Cambridge Research Systems is used [43]. This device, called BlueGain, has two differential analogue input channels to record the horizontal and vertical movements of the eyes. The signals recorded by the two channels are amplified by instrumental amplifiers. Then, these signals are digitized using a 16-bit ADC at 10 kHz. The signals are sent raw to the computer, where the processing is performed. This bioamplifier is provided with a test software application for signal display and filtering (Figure 3).

### 4.2. Software Application

As shown in Figure 4, the software application consists of three modules: signal processing, feature extraction, and the graphical user interface (GUI). Before the signal processing, several Matlab functions included in the BlueGain Toolbox are used to connect the device and obtain the data in the software application. These functions are the following:

- BlueGainRequestData requests a packet of data from BlueGain.
- BlueGainRetrieveData retrieves data from BlueGain.
- BlueGainResetSampling resets the sampling of BlueGain and clears the buffer.
- crsFindSerialPort finds the serial port of the computer where the Bluetooth device is connected.
- crsOpenSerialPort opens the port that was previously found by the former function.

These functions are called by means of the function EOG Callback, which is part of the Matlab source code, and is provided together with the article as Supplementary Material.

#### 4.2.1. EOG Signal Processing

The EOG signal has a major artefact problem. It is small in amplitude and consists of very low frequencies. Removing artefacts without losing original features of the signal is a challenging task, one that is an active area of research. In this work the signal is digitally filtered to reduce the noise that is not removed by the hardware.

**Figure 5:**
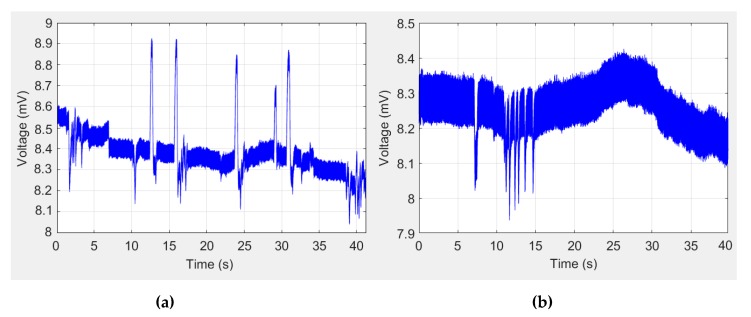
Examples of EOG signals before being digitally processed in the computer: (**a**) signal in which the peaks of the saccades stand out on the SEM of tracking and fixation; (**b**) signal in which only fixation micromovements are identified.

Figure 5 shows two examples of signals received in the computer before being digitally processed. Figure 5a shows a signal in which the peaks of the saccades stand out on the SEM of tracking and fixation, whereas only the fixation micromovements can be identified in Figure 5b. A 30-Hz low-pass ninth-order Butterworth filter is implemented using MATLAB’s signal processing toolbox. Once the signal noise has been removed, to identify the saccades, the derivative of the signal is calculated using the approximation of finite central differences equation:

(1) $$f^{\prime }\left(x_{0}\right)=\frac{f\left(x_{0}+h\right)-f\left(x_{0}-h\right)}{2h}$$

where $x_{0}$ is the point at which the derivative is made and h is the distance to the previous and subsequent points. Next, the peaks of the signal are compared with a threshold value Th to calculate how often saccadic movements occur (Equation (2)). The value of the parameter Th is calculated empirically by initial tests in healthy subjects:

(2) $$\{\begin{array}{l} x^{\prime \left(t\right)}\geq Th\;\to Saccade\\ x^{\prime \left(t\right)}<Th\;\to No\;saccade \end{array}$$

#### 4.2.2. Feature Extraction

From the observation of REM and SEM on moving and fixed stimulus, three evaluation parameters can be defined: rate of saccades, deviation, and response delay. The rate of saccades (*ROS*) is the number of saccades generated during the test. This parameter is calculated by Equation (3), where N refers to the number of times the threshold value is exceeded and T is the duration of the test (45 s):

(3) $$ROS=\frac{N}{T}$$

A media filter considering the last 100 samples is then applied. The result is a smoothed signal in which SEM is represented by signals close to a constant of zero value, whereas REM appears as peaks and can be clearly seen when unwanted movements occur.

During the first 5 s of the test, the stimulus is still, and the patient must look at it to calculate the deviation parameter, which is the error of amplitude in the direction of the gaze with respect to the position of the stimulus. This error can be positive (advance) or negative (delay). In Figure 2, this deviation is represented by the angle ϕ (Equation (4)). The value of the reference angle under which the test is performed, θ, is zero. The observation angle of the patient, φ, can be estimated by Equation (5), where V is the amplitude of the signal and S is the sensitivity. As discussed in the previous section, a sensitivity of 16 µV/° is considered:

(4) ϕ=φ−θ

(5) $$\varphi =\frac{V}{S}$$

The response delay (*D*) is the time of the patient’s saccadic reaction to a visual stimulus. This parameter is calculated by Equation (6), where n refers to the number of samples between the start of the stimulus movement and the corresponding saccade. $T_{s}$ refers to the data stream to the host computer, which is 1 ms according to the specifications of the hardware device:

(6) $$D=n\times T_{s}$$

#### 4.2.3. Graphical User Interface

**Figure 6:**
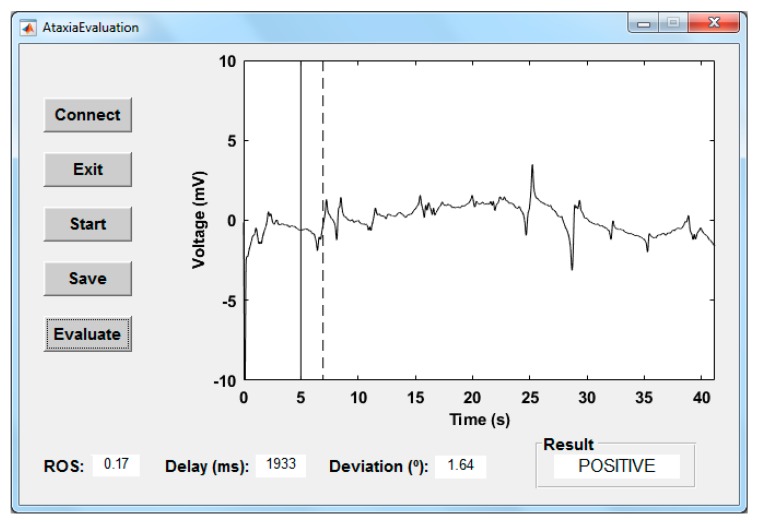
Graphical user interface of the software application developed.

**Figure 7:**
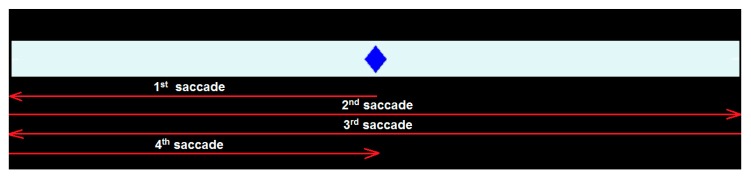
Representation of the stimulus movement and the associated saccadic eye movements.

To evaluate the behavior of eye movements, a graphical user interface (GUI) was developed using MATLAB R2018b (Figure 6). This software application called AtaxiaEvaluation is available as Supplementary Material to this article. After pressing the Connect button, the hardware device starts sending signals to the computer via the Bluetooth connection. By pressing the Start button, a stimulus, in the form of a rhombus (Figure 7) and implemented as a video, will be shown on the screen. This will remain static for 5 s (vertical marker in Figure 6) during which the deviation parameter will be calculated. The moment in which the eye responds to the movement of the stimulus is represented by the dashed marker in Figure 6. Subjects must follow the movement of the stimulus with their gaze. Their responses are automatically stored in ASCII-formatted files by pressing Save button in case it is necessary to visualize again the data obtained or that these must be reprocessed.

According to the value of the three parameters exposed in Section 4.2.2, the system will classify two possible behaviours of the individuals: possible ataxia (positive) and healthy (negative). The parameters have the same weight in the decision criteria, and the three values obtained should be within those considered normal according to the designed test and those available in the literature.

During the test, the stimulus makes four changes of direction on the horizontal axis (as shown in Figure 7), the subject is expected to make as many saccadic eye movements. If a margin of only two possible involuntary saccades is allowed, a *ROS* equal or less than 0.13 (6 saccades) for a healthy person is set. On the other hand, the time delay in vision is at least 40 ms, being the mean reaction time to detect visual stimuli approximately 140–180 ms, according to the literature [44,45]. Other studies have documented that the perceptions for visual stimulus appearances may be slightly higher at around 180–200 ms [46,47]. There is a study that indicates that the time delay can even reach 300 ms [48]. So, a response delay less than 300 ms for a healthy person is established in this work. Lastly, during sustained fixation, eye micromovements have maximum amplitude of 1.5° [47,49,50]. As the EOG technique has a typical accuracy of approximately ±1.5°, a gaze deviation threshold of 3° is set. If one of the three parameters is exceeded, the result is considered positive.

Finally, by pressing the Evaluate button, the result of the evaluation will be shown. The processed EOG signal and the values obtained in the three parameters can be examined to see which have had an influence in the case of a positive result.

## 5. Experimental Results

The proposed system was evaluated by 10 subjects between 32 and 64 years old (mean = 51.4, SD = 9.9). Five of the subjects have already been diagnosed as suffering from some type of ataxia, and the other five subjects are neurologically healthy. Table 1 shows the characteristics of each of the subjects who participated in the tests. Each of them performed three tests to verify the consistency of the results obtained. The study was conducted in accordance with the Declaration of Helsinki and approved by the University of Oviedo’s Institutional Ethics Board. All participants signed a consent form before starting the tests.

### 5.1. Experimental Setup

**Figure 8:**
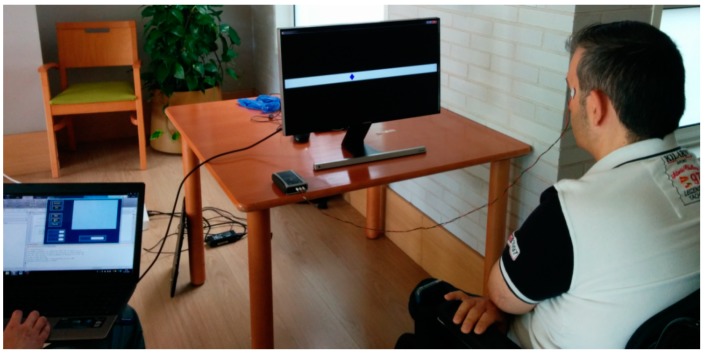
Setup for testing the application software with the BlueGain bioamplifier on the table.

All volunteers were placed in a chair approximately 60 cm from the interface and oriented in such a way that the stimulus was in the same horizontal plane as their eyes, as shown in Figure 8. The designed interface was presented on the screen (S24D590L 23.6”, Samsung, Seoul, Korea), which has a display area of about 540 × 343 mm.

Under this arrangement, ±32.4° eye movement amplitudes are obtained when the stimulus is at the left and right ends of the screen. Volunteers were told to keep their heads as still as possible. It is advisable to use a head fixation device to facilitate this. Neuroline 70010-K/C Ag/AgCl surface electrodes (Ambu, Ballerup, Denmark) of 20 × 15 mm dimension with a light-duty cable and a 1.5 mm touchproof safety socket were used. They were positioned after the skin had been cleaned with alcohol to remove facial impurities and reduce skin impedance. The electrodes were placed as shown in Figure 4.

**Figure 9:**
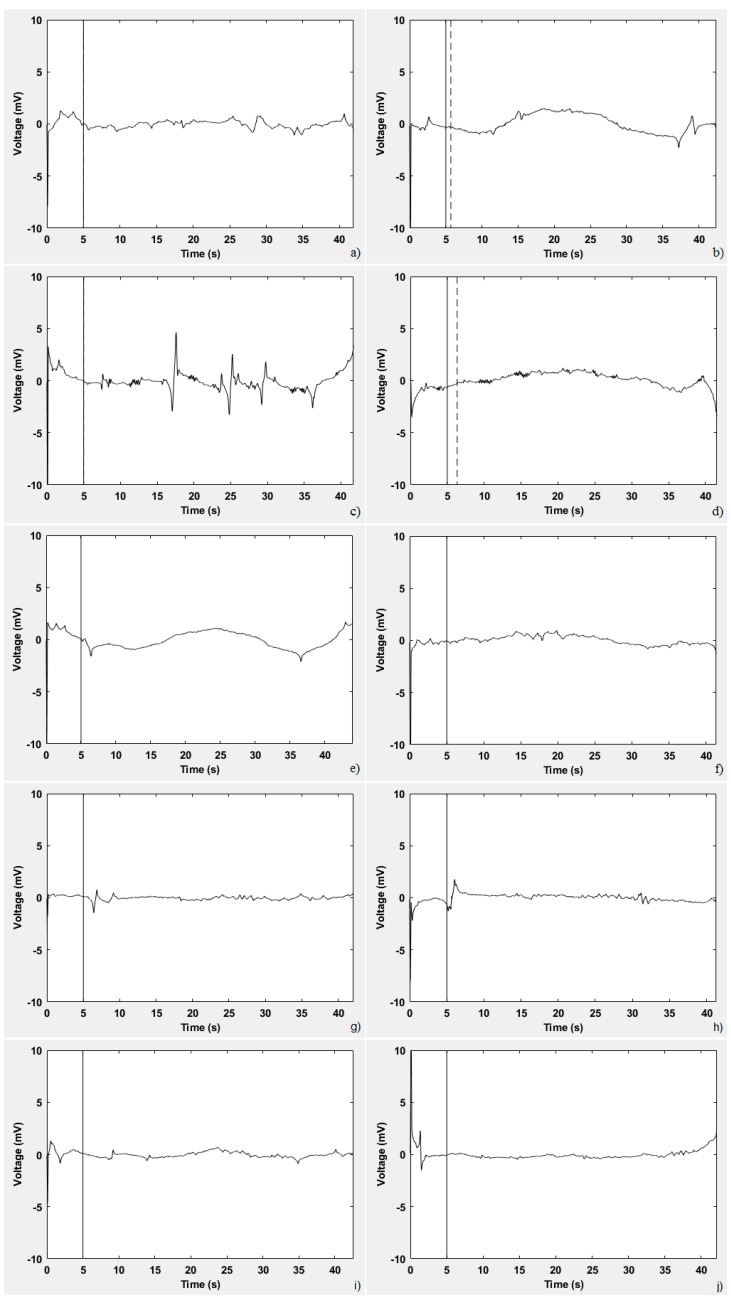
EOG-processed signals obtained by the ten subjects (a–j in Table 1) involved in the tests.

Figure 9 displays the processed signal obtained by the ten subjects presented in Table 1. Table 2 presents the values obtained in the three parameters under analysis (highlighting the abnormal) as well as the result of the evaluation for each of these ten subjects. Figure 9a displays the signal obtained by subject ‘a’. He has difficulty in fixing his gaze on the stimulus; hence, the positive result of the evaluation, as reflected in Table 2. Figure 9b corresponds to a 54-year-old man who suffers from an ataxia due to an injury caused by an accident, which presents as an abnormal delay and deviation in response to the stimulus. The signal of a woman who suffers from Friedreich’s ataxia is shown in Figure 9c. The signal is close to zero, but involuntary saccades are observed. It is expected that, if ataxia were more advanced, the number of saccade movements would be greater. Figure 9d,e correspond to two men (‘d’ and ‘e’ in Table 1) who suffer from a hereditary ataxia of unknown type, which presents as an abnormal delay and deviation, respectively, in response to the stimulus. In contrast, in Figure 9f, a signal close to zero, without peaks and significant deviations or delays, was obtained by the subject ‘f’. Because the three parameters analysed are within the established ranges, the result of the evaluation is negative. A similar result was obtained by the four other healthy subjects.

### 5.2. Evaluation of Results

The evaluation of the designed application is made by means of three metrics: sensitivity, specificity, and accuracy. The sensitivity (Equation (7)) indicates the ability of the system to accurately identify positive cases, that is, to identify ataxia when the individual really has the condition. The specificity (Equation (8)) measures the proportion of negatives that are correctly identified, that is, to conclude that an individual does not have ataxia. Accuracy is the proportion of correctly identified cases, as described by Equation (9):

(7) $$Sensitivity\;\left(\%\right)=\frac{TP}{TP+FN}\times 100$$

(8) $$Specificity\;\left(\%\right)=\frac{TN}{TN+FP}\times 100$$

(9) $$Accuracy\;\left(\%\right)=\frac{TN+TP}{TN+FP+FN+TP}\times 100$$

where:

- TP (true positive) represents the number of positives generated when an evaluation result is correct.
- FP (false positive) represents cases where the system identifies as positive an individual who does not have ataxia.
- TN (true negative) represents a case where a normal eye movement is performed, and therefore the system does not identify ataxia.
- FN (false negative) refers to an individual who does have ataxia, but it is not identified by the system.

In the three tests performed by the 10 subjects (30 tests in total), 13 TPs, two FPs, 12 TNs, and three FNs were obtained. Thus, 81.3% sensitivity, 85.7% specificity, and 83.3% accuracy were obtained. These results give viability to the idea proposed here and encourage further work in this regard. The most reliable form of diagnosis is through a genetic analysis, which provides an accuracy of 99.9%. These tests may cost several hundred dollars apiece, posing a financial barrier in many cases [1]. Besides, it is necessary to perform a genetic test for each type of ataxia and because of the difficulty of knowing the type of ataxia of each test subject.

From the results obtained, it can be said that the EOG and, therefore, the method used to detect ataxia do not depend on the patient’s age or gender. The signals measured in women, regardless of age, seem slightly more stable than those of men, although it cannot be determined with precision whether this always happens because the study involved only a small group of people. However, it can be concluded that there are significant differences between the recorded EOG for healthy individuals and those affected by ataxia. The processed signal will have an almost-constant value close to zero for people who do not have ataxia.

To obtain a more accurate evaluation, other parameters such as the calculation of the signal power could be considered. The power of the EOG signal from all subjects was obtained. The average power is 18.58 W for healthy people and 3.19 W for ataxic ones. However, these values depend on the amplitude of the signal squared which varies with each subject. A variance of 317.13 and 7.73 in healthy and patients affected by ataxia was obtained respectively. Therefore, in principle, it is not possible to establish a relationship between the power of the EOG signal and a possible ataxic disorder. More tests should be necessary to consider the power as a relevant parameter.

The test results yielded several other observations on which we can continue to work. The most important is that the quality of the acquired signals depends largely on electrode placement. Electrodes should be placed above the bony structure to avoid any subject discomfort that can increase the number of blinks and to minimize EMG interference. EOG signal acquisition also showed low tolerance to head movements. In this sense, an ergonomic system that integrates dry electrodes and can be easily attached would be advisable. In the literature, several designs based on conventional glasses or similar ones designed to integrate the electrodes or even the acquisition device can be found [51,52,53]. Moreover, the corneo-retinal potential is not fixed. It varies diurnally due to lighting, fatigue, and age variables that must be considered.

## 6. Conclusions

This paper reports an affordable ataxic disorders evaluation method based on the processing of EOG, which could be used to replace the traditional naked-eye method. The EOG, combined with a computer application, may be a useful for testing a patient’s eye coordination and identifying whether the subject might have some type of ataxia. The results obtained show the feasibility of the proposed method as a promising clinical assessment modality for performing an objective evaluation. However, signal processing and parameter setting improvements are necessary.

For physicians, the use of this interface is simple and requires minimal training. People who are affected by ataxia might present additional symptoms. In this sense, the aim of the proposed method is to be a complementary evaluation tool, but it is not conclusive because a disease is generally not related in a biunivocal way to a symptom. Each symptom or finding in an exploration presents a probability of appearance in each disease. The clinical diagnosis requires considering more aspects of analysis, such as the anamnesis or complementary explorations.

In future studies, the movement performed by the stimulus, based on which the evaluation is made, could be replaced by another or in both axes. On the other hand, other parameters that complement the exposed evaluation criteria could be considered. A greater number of different tests would allow a more reliable evaluation. A better knowledge of EOGs of patients with different types of ataxia disorder would also improve the identification of patients and make it easier to distinguish the type of ataxia that they suffer.

## Figures and Tables

**Table 1:** Characteristics of the Subjects Involved in the Tests.

Ataxia Patients				Healthy		
Subject	Age	Gender	Origin	Subject	Age	Gender
**a**	46	Male	Hereditary—Unknown	f	41	Female
**b**	54	Male	Non hereditary—Accident	g	57	Male
**c**	60	Female	Hereditary—Friedreich	h	32	Male
**d**	64	Male	Hereditary—Unknown	i	58	Female
**e**	56	Male	Hereditary—Unknown	j	46	Female

**Table 2:** Values Obtained in the Three Parameters under Analysis (Highlighting the Abnormal) as well as the Evaluation Result.

Subject	*ROS*	Deviation (^°^)	Response Delay (ms)	Result
**a**	0.08	21.03	156	Positive
**b**	0.13	5.87	615	Positive
**c**	0.20	2.08	149	Positive
**d**	0.08	1.60	1340	Positive
**e**	0.13	3.32	128	Positive
**f**	0.08	0.45	135	Negative
**g**	0.11	0.71	174	Negative
**h**	0.11	2.26	153	Negative
**i**	0.08	1.82	139	Negative
**j**	0.08	0.94	181	Negative

## Supplementary Materials

The following are available online at https://www.mdpi.com/1424-8220/19/17/3756/s1, Software application: AtaxiaEvaluation.exe, Source code of the software application: AtaxiaEvaluation.m.
