# Supplementary figures and images for: An Affordable Method for Evaluation of Ataxic Disorders Based on Electrooculography
Source: Sensors (Basel). 2019 Aug 30;19(17):3756. doi: 10.3390/s19173756 (PMC6751503; doi:10.3390/s19173756)

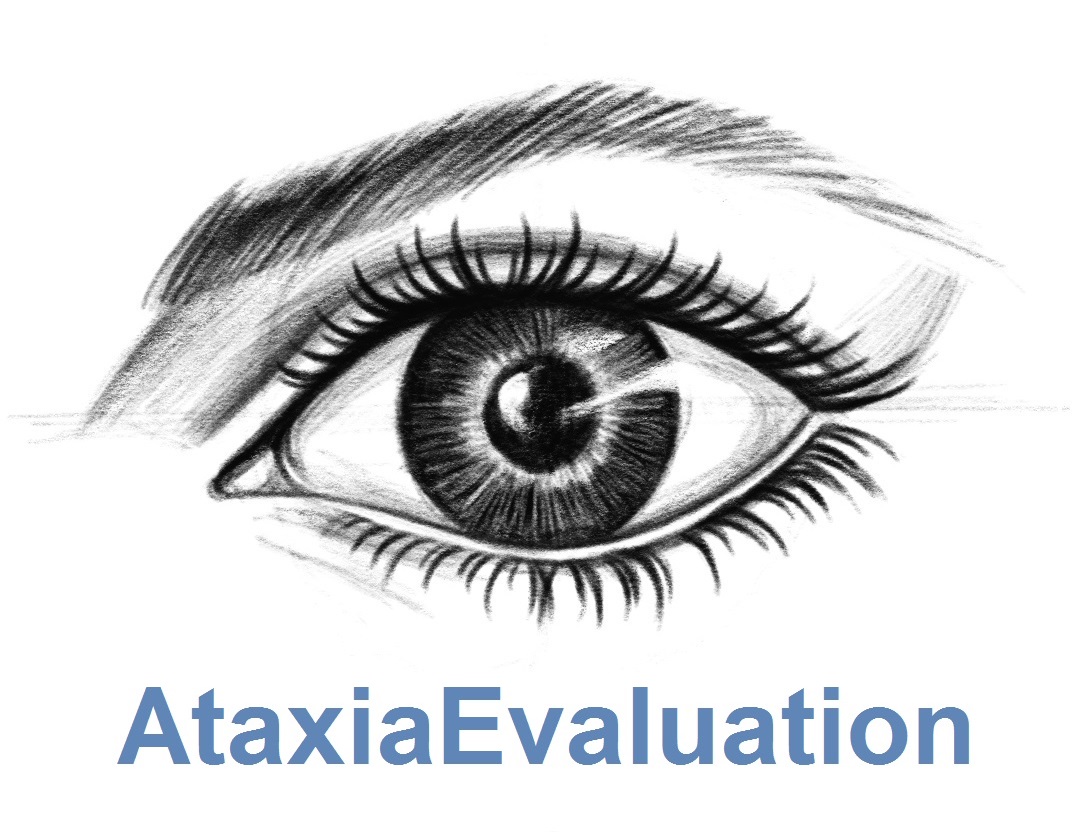

Supplement: Supplementary file 1 [file sensors-19-03756-s001.zip › Supplementary Files/AtaxiaEvaluation App/splash.png]
